# Development of double blind gluten & casein free (GFCF) test foods for autism trial

**DOI:** 10.1186/1745-6215-14-S1-P22

**Published:** 2013-11-29

**Authors:** Elaine McColl, Sandra Adams, Nicola Burton, Anna Cutress, Ashley Adamson, Gillian Baird, Anne O'Hare, Ann Le Couteur

**Affiliations:** 1 Newcastle University, Newcastle upon Tyne, UK; 2 Northumbria Healthcare NHS Foundation Trust, North Shields, UK; 3 Guy's and St Thomas' NHS Foundation Trust, London, UK; 4 Edinburgh University, Edinburgh, UK

## Background

Autism is a severe long-term neurodevelopmental disorder. Within this population, there is a need to investigate the efficacy of GFCF diets in an adequately powered, randomised controlled trial; double blinding to the removal of gluten and/or casein is desirable but complex to accomplish. We tested the feasibility of producing test foods to achieve.

## Methods

A company, developed a range of test foods - muffins, porridge, batter mix, lemon & almond cookies and chocolate krispie bars, containing the estimated average habitual child intake of gluten (5g) and/or casein (5g): *Gluten and casein added* (*GACA*)*; Added casein only* (*GFCA*)*; Added gluten only* (*GACF*)*. No gluten or casein added* (*GFCF*)*.* We aimed to recruit 60 children (3-6 yrs) with autism and to randomly allocate them to receive test foods from one the above groups (labelled A-D to maintain blinding) for 28 days alongside normal diet. Outcomes were measured by food consumption diaries, a test foods acceptability questionnaire and telephone interviews with a sub-set of parents.

## Results

A total of 52 children were recruited; 3 families withdrew due to food refusal. Muffins and chocolate krispies were most readily accepted. Rice porridge was least well liked. Children receiving GACF and GACA test foods were more likely to consume at least half a portion of food when offered.

## Conclusion

Families demonstrated a high level of motivation and commitment. The planning and execution of this study were challenging but a range of test foods suitable for young children with autism have been produced.

